# Editorial: Targeting metabolism of cancer cells and host to overcome drug resistance: Preclinical and clinical studies

**DOI:** 10.3389/fonc.2023.1154661

**Published:** 2023-02-10

**Authors:** Lishun Wang, Hanchen Xu, Yadi Wu

**Affiliations:** ^1^ Center for traditional Chinese medicine and gut microbiota, Minhang Hospital, Fudan University, Shanghai, China; ^2^ Institute of Digestive Diseases, Longhua Hospital, Shanghai University of Traditional Chinese Medicine, Shanghai, China; ^3^ Department of Pharmacology & Nutritional Sciences, Markey Cancer Center, the University of Kentucky, College of Medicine, Lexington, KY, United States

**Keywords:** cancer metabolism, drug resistance, gut microbiome, omics, metabolite

This collection contains seven articles published in “Frontiers in Oncology” between September 2, 2022, and December 12, 2022, and broadly focuses on the amino acid metabolism in cancer cells, the metabolites of microbes in the gut of the host, and the methodology of omics in cancer metabolism, all of which shed a light on the road to overcoming drug resistance in cancer therapy.

Cancer is one of the leading causes of death in most countries worldwide (1). Metabolic perturbation is known as a hallmark of cancer and abnormal metabolic alterations play a critical role in the occurrence and development of cancers (2). Moreover, the metabolic alterations in cancer enable cancer cells’ drug resistance in cancer therapy (3). Metabolic factors have recently been suggested as one part of the important targets for the development of novel, combinatory drugs to overcome resistance to chemotherapy, target therapy, and immunotherapy (4). In addition, host metabolic factors, such as metabolites derived from commensal gut microbiota, have also been recognized as modifiers of the cancer microenvironment and have been targeted for therapeutic gain in cancer (5, 6).

The metabolites derived from amino acids have been recognized as modifiers of the cancer microenvironment and targets for cancer therapeutics. As a non-essential amino acid, glutamine can be synthesized by cells. Glutamine metabolism contributes to the growth and proliferation of mammalian cells as well as tumor cells. Yang et al. summarized the role of glutamine metabolism in ovarian cancer cell proliferation, invasion, and drug resistance. They also discussed the role of glutamine in protein synthesis and in purine and pyrimidine synthesis as primary nitrogen donors. In addition, they reviewed studies that observed that glutamine-addicted tumor cells depend on glutamine for survival. Most interestingly, combining platinum-based chemotherapy with the inhibition of glutamine metabolic pathways may be a new strategy for treating ovarian cancer, especially drug-resistant ovarian cancer.

Branched-chain amino acid metabolism affects systemic metabolism in cancer cells. Branched-chain amino acid transferase (BCAT) is an enzyme that catalyzes the transamination of three branched-chain amino acids to branched-chain keto acids. Nong et al. reviewed the potential roles of BCAT in different cancer development and treatments. They also discussed the BCAT as the target of the proto-oncogene c-Myc. Furthermore, they emphasized that BCAT usually promotes cancer proliferation and invasion by activating the phosphatidylinositol 3-kinase/protein kinase B/mammalian target of the rapamycin pathway as well as Wnt/β-Catenin signal transduction.

Cornett et al. summarized another well-known gene, GAPDH, involved in glycolysis. Recently, GAPDH was found to have diverse localizations and its role is largely dependent on its cellular location and interaction partners. They discussed the membrane-associated function of GAPDH in stimulating glucose uptake in neuroblastoma and the nuclear complex of GAPDH in DNA repair, which demonstrated its potential role in cancer metabolism, treatment, and drug resistance.

The perceptions of cancer metabolism are driven by technological and methodological advances in omics. Genome sequencing has been widely used in clinic cancer target therapeutics. Li et al. conducted next-generation sequencing for a rare case of secondary tumor of the ovary from the liver and identified a BRCA2 mutation. Therefore, they treated the patient with polyadenosine diphosphate-ribose polymerase inhibitor olaparib after the administration of surgery. The patient has achieved nearly 2-year survival and lives a relatively normal life with good quality. Meanwhile, significant amount of information is contained in the public cancer genomics database, waiting for deep mining to provide clues for cancer therapeutics. To this end, Li et al. analyzed the data from the Genomics of Drug Sensitivity in Cancer (GDSC) database and found that cuproptosis-related genes are associated with the development, tumor microenvironment, and prognosis of lung adenocarcinoma. They also provided a scoring system based on these cuproptosis-related genes to predict the efficacy of targeted drugs and immune response. These findings may indicate the potential roles of copper metabolism in cancer biology and provide a new path for the assessment of cancer prognosis and therapeutics.

Spatial metabolomics offers an opportunity to demonstrate the drug-resistant tumor profile with metabolic heterogeneity and poses a data-mining challenge to reveal meaningful insights from high-dimensional spatial information. Zhang et al. discussed the latest progress, with a focus on currently available bulk, single-cell and spatial metabolomics technologies and their successful applications in cancer drug resistance. They summarized the underlying metabolic mechanisms, including the Warburg effect, altered amino acid/lipid/drug metabolism, generation of drug-resistant cancer stem cells, and immunosuppressive metabolism. The perspective of how the spatial metabolomics approach (integrating spatial metabolomics) could be further developed to improve the management of drug resistance in cancer patients is expounded.

More recently, the gut microbiome has demonstrated great influence on cancer formation, prognosis, and treatment *via* their metabolites. Huang and Mao discussed the effects and the underlying mechanisms of the gut microbiome and microbial-derived metabolites in cancer development and treatment. They reviewed research on gut microbiome intervention by transplantation of fecal microbiota from healthy volunteers to cancer patients, which can suppress the carcinogenesis, or augment the therapeutic effect on the tumor through the related metabolites, suggesting targeting gut microbiome will be a new approach to improve the efficacy of tumor prevention and treatment.

Overall, our Research Topic highlights the ongoing challenges in the field of amino acid metabolism in cancer cells, the metabolites of microbes in the gut of the host, and the methodology of omics in cancer metabolism. This knowledge will ultimately contribute to a better understanding of the role of metabolism in tumorigenesis and advance the translation of these findings to overcome drug resistance in cancer therapy.

## Author contributions

All authors listed have made a substantial, direct, and intellectual contribution to the work and approved it for publication.
